# Coping with suicide loss: a qualitative study in primary health care

**DOI:** 10.1017/S1463423622000263

**Published:** 2022-07-25

**Authors:** Kadri Suija, Liis Rooväli, Merli Aksen, Heti Pisarev, Anneli Uusküla, Raul-Allan Kiivet

**Affiliations:** 1 Institute of Family Medicine and Public Health, Faculty of Medicine, University of Tartu, Tartu, Estonia; 2 The Centre for Applied Social Sciences, University of Tartu, Tartu, Estonia

**Keywords:** bereavement, consultation, death and dying, mental health, primary care, suicide

## Abstract

**Aim:**

To analyze how people cope with suicide loss and the implications for primary health care.

**Background:**

Previous studies have shown that primary health care will often be an initial source of support for those bereaved by suicide.

**Methods:**

We included adult persons who were ready to talk about a suicide completed by a person they knew well (family member or close friend). Participants were recruited via mixed media (television, radio, print, social media, etc.). Altogether, we conducted 37 individual interviews, which were recorded using a dictaphone and lasted from 46 to 158 min. The interviews were transcribed verbatim and analyzed using a content analysis method. The interviewees were mostly women (*n* = 27) and family members (*n* = 28) of a person who had died by suicide during the years 2012–2018.

**Findings:**

We identified two main themes in the data: supporters and barriers in support. Coping with suicide takes time, and support was mostly found among friends and family. Support from GPs was mentioned in the context of diagnosing medical problems and prescribing medicines. Respondents indicated that feeling ashamed and a lack of trust impeded their willingness to seek help from their GP. Unmet needs among the bereaved may increase their risk of diminished mental health outcomes. Thus, primary health care practitioners may have a substantial opportunity to support those who are bereaved by suicide.

**Conclusion:**

Primary care providers have an opportunity to provide bereavement support among their patients. Continuing medical education regarding the needs of the bereaved and a coordinated approach among primary care practitioners may be useful to proactively identifying and supporting those in need.

Suicide is a complex phenomenon with various triggers. Despite the abundance of research and prevention plans, suicides continue to claim too many lives. It is estimated that every 40 s someone dies by suicide somewhere in the world (WHO, 2014). The average standardized death rate due to suicide is 10 per 100 000 inhabitants across Europe and higher in some countries, such as 14 per 100 000 inhabitants in Estonia (Eurostat, 2017).

One key factor in prevention of suicide is an appreciation of who is at risk (WHO, 2014). In the extensive literature about risk factors for suicide, one is bereavement by suicide (Qin *et al.,* 2002; de Groot and Kollen *et al.,* 2013; Pitman *et al.,* 2016). Studies have shown that suicide death bereavement differs from bereavement caused by natural death (Pitman *et al.,* 2016). One recent study found that every death by suicide affects approximately 135 people who are bereaved by that loss; people struggling to cope with bereavement often need professional help (Cerel *et al.,* 2019). On the other hand, individuals bereaved by suicide may avoid actively seeking help to do stigma (Pitman *et al.,* 2018). To improve linkage to care among those bereaved by suicide, interactions with the health care system should be identified.

Studies have shown that 20%–76% of people who completed suicide visited their primary care providers a month before and 57%–90% a year before suicide (Luoma *et al.,* 2002). This finding underscores the importance of studying suicide risk factors that could be identified by primary care practitioners and opportunities to help those at risk within the practice. We also know that people bereaved by suicide continue to visit their primary health care practitioner in the days and months surrounding their loss. Although the literature is still emerging on the role of primary care in suicide bereavement, early data are promising. One recent study evaluated parents’ experiences of support from primary care following the suicide death of their son or daughter and found that these parents reported the GP to be an important source of support and knowledge (Wainwright *et al.,* 2020). Similarly, other studies have presented that despite some negative experiences, most of the people found that health professional help was paramount (Pitman *et al.,* 2018).

The effect of suicide bereavement is not limited to the family of the deceased (Pitman *et al.,* 2016). Therefore, we should also gather sufficient data to describe the bereavement needs and experiences among those who suffer a loss to suicide outside the immediate family. Our aim was to study how people cope with suicide loss and the implications for primary health care.

## Methods

We included adult persons who able to speak the Estonian language and who were ready to talk about a loss to suicide completed by a person they knew well (eg, husband, wife, parent, grandparent, child, close friend, etc.). Participants aged 18 years or more were recruited using a broad sampling method by invitation outreach shared via media (TV, radio, newspapers, webpages, mailing lists) and social media (Facebook, Twitter). Potentially interested participants contacted the research team by phone or by mail and were provided further information about the study.

Altogether, 72 persons contacted the study team during the three months’ study period. Of these, 35 were excluded for various reasons: some wanted to discuss this topic in general, some were worried about their mental health and thus had needs beyond our capacity to immediately support (in this case, the respondent was given information about available services), some had experience with suicide attempts but not suicide death or they did not know the case well enough (eg, suicide was completed by their friend’s friend or their acquaintance). People who had been bereaved less than 10 months or more than 10 years ago were excluded because our background research suggested that participation too early may be too distressing; on the other hand, when too much time has passed recollection may be difficult and inaccurate (Rooväli *et al.,* 2018).

One researcher (LR) who is also a medical doctor talked by phone with all persons who contacted with the study team. For participants who were eligible for our study and who confirmed they would like to participate, a convenient time and place to conduct the individual interview was agreed upon. For participant who was not eligible for our study was given information on the content of this study and available services for help if needed.

One-to-one interviews were used for data collection. The interviews were performed at the University of Tartu campus, at the interviewee’s home or work, private rooms of a public library or in public places (eg, café) according to the preferences of respondents. One interview was performed via Skype and two by telephone. The interviews were conducted by three researchers (LR, MA or KS). Two of the researchers are medical doctors, and one has significant professional experience in conducting interviews; all three researchers are female.

All participants signed the written voluntarily informed consent to take part in the study; they also received written information about the study and information on available services for help if desired. Based on the feedback of the interviewers despite the emotional topic, the interviews went without problems. None of the participants contacted the study team later with any negative comments regarding his/her interview; to the contrary, all participants reported a sense of gratitude for the opportunity to discuss their difficult experience.

Table 1 presents the main interview topics. The semi-structured interview guide was developed by the authors following a literature review. Open questions were used for encouraging discussion and for enabling respondents to express their personal experiences. Two topics were covered in the interview guide: (1) the life and experiences of the person who completed suicide; and (2) the life and experiences of the bereaved person.

**Table 1.** Interview topics

Questions related to the person who committed suicide His/her lifestyle and family situation, education and work, hobbies and free time, health problems and used medications, alcohol and drugs use, previous suicide thought(s) and/or attempt(s) Questions related to the interviewed person His/her feelings and thoughts about the suicide of the close one bereavement process and coping with the suicide ideas about how to prevent suicide and support people related with it

The interviews were recorded using a dictaphone and lasted from 46 to 158 min (mean 90 min). The interviews were transcribed verbatim and analyzed using inductive content analysis method (Malterud, 2001; Hsieh and Shannon, 2005; Braun and Clarke, 2006). Analysis was performed collaboratively with all researchers. We defined the units, categories as well as rules of analysis to find out answers to the study questions how people cope with suicide loss and the implications for primary health care. Firstly, two researchers (KS and LR) read the transcripts to get an overview of the interviews. Secondly, they identified meaning units, which were then categorized and labeled as codes. Thirdly, the codes were sorted into groups sharing similar content. This was constantly compared and cross-referenced between transcripts. All interpretations of the data were regularly discussed between the researchers and interviewers to agree upon the broader categories. Unanimous consent was required of all authors prior to confirming a theme and to draw conclusions.

Study reporting was based on the COREQ criteria and recommended standards (Tong *et al.,* 2007; O’Brien *et al.,* 2014).

The Ethics Committee of University of Tartu approved this study.

## Results

### Study sample

Altogether, we conducted 37 individual semi-structured interviews; upon inspection of the data collected, we found the information gathered was sufficiently saturated for analysis. We did not carry out any repeat interviews.

The interviewees were mostly (*n* = 28) family members of the person who had died by suicide. We conducted interviews with parents (*n* = 10), with a husband/wife or a partner (*n* = 8), with siblings (*n* = 7), with grown-up children (*n* = 2) and with grown-up grand-children (*n* = 1) of the person who had died by suicide. Of the participants, 9 persons reported that the suicide victim was their close friend. Most of the interviewees were women (*n* = 27), and in 31 of the 37 losses, the person who had died by suicide was male. The persons who completed suicide were 16–82 years old during their suicide, and the suicide had happened in years 2012–2018.

In this paper, we will present analysis of the questions related to the interviewed person’s experience. We identified two main themes in the data: supporters and barriers in support. Data extracts are provided to illustrate these themes.

### Supporters

All interviewees reported that coping with suicide has been difficult and has taken time. Some participants (mostly parents) admitted that they have not got over it at all and are still struggling to cope with the loss.

‘I left a job and I have never gone back to work after that, I do not have strength to go to work again. I suffer from depression, take antidepressants now’. (a parent, interview 17)

We asked the participants what had helped them during the bereavement. Most often they mentioned help from close persons (friends or family members), with whom it was possible to talk, someone who would listen and whose presence was important.

‘I was able to discuss this tangle with help my family and friends and this helped me in grief’. (a friend, interview number 20)

Regarding professional support, participants reported that they received relevant help from psychologists and psychiatrists. Some people mentioned that they still rely on a psychologist’s help, specifically commenting on the importance of receiving necessary help quickly.

Support from a GP was mostly mentioned in relation to diagnosing depression and prescribing medicines. Several interviewed persons admitted they used or continue to use antidepressants.

‘My general practitioner performed a test and it showed something, like depression and then he prescribed me something, I do not remember the name anymore’. (a partner, interview number 25)

Interviewed people reported that general information related to suicide, death and following procedures was helpful. It was mostly received from authorities (eg, police) and less from primary health care providers.

Quite many respondents reported that they found reading self-help books to be useful, as was participating in support groups with people with similar experiences. Some people found that concentrating on everyday responsibilities and work was actually the most helpful during the bereavement.

‘What has helped me…, mostly my work’. (a parent, interview number 18).

### Barriers to support

Participants reported that coping with suicide was complicated when they did not have the possibility to openly talk about it. The latter was sometimes related to protecting important others.

‘You see, I do not have anyone to talk about it. I do not want to talk with my children either, they are also broken’. (a parent, interview number 8)

The interviewees pointed out that professional support was rare and mostly was offered to direct family members and not so much to other bereaving people.

‘I do not have the opportunity to go to a support group’. (a sibling, interview number 28)

Participants reported various barriers which impeded asking for help from the GP. The reasons were mostly related to shame but also included a lack of trust in the GP.

‘I think that lot of people feel a bit ashamed /…/ to go to the general practitioner and say that I have, I do not know, depression or something. Not everyone has a good physician also, or the general practitioner does not care or is not interested, and you just do not want to go to the general practitioner’. (a partner, interview number 12)

Some people did not consult with their GP at all and some mentioned that they asked but experienced a negative attitude from the GP.

‘I asked my general practitioner… but she said there is no money for a psychologist. Then I got a referral with the help of my friend’. (a parent, interview number 10)

The contacts with the GP were irregular, often resulting in the bereaved individual having to find support alone and not receiving appropriate help at the right time. Some people also considered death to be a normal life event which does not need medical help and professional support.

‘No, /./, even, I have had earlier also difficult hits, when my father died’ (a friend, interview number 9)

‘It makes me, feel even worse, if I have to talk about it again and again and again’ (a grandchild, interview number 19)

## Discussion

Based on the interviews with persons bereaved by suicide, coping with suicide takes time. Most support is found from friends and family. Support from one’s primary health care provider was mentioned in relation to diagnosing medical problems and prescribing medicines. Moreover, interviewed people mentioned shame and a lack of trust as important barriers to asking for support from their GP. These negative experiences with general practice may be specific to their bereavement process or may be related to previous negative experiences. Quite often, our participants had the same GP as the suicide victim, which may suggest some perceived transference/countertransference – feelings, expectations and beliefs a patient or a physician brings to the doctor-patient relationship and which may influence the rapport.

Communicating with those in bereavement requires specific skills, and not all GPs are sufficiently trained or have the confidence to interact in this way. For example, previous research has found that some GPs lack the knowledge and skills to help people bereaved by suicide (Pitman *et al.,* 2018; Wainwright *et al.,* 2020; Ross *et al.,* 2021). On the other hand, research has also found that a GP who can offer special support services for those going through this unique loss and grief process is valued by patients (Pitman *et al.,* 2018; Wainwright *et al.,* 2020; Ross *et al.,* 2021). Mixed, conflicting emotions, shame, distrust, feelings of guilt and stigma are common among people bereaved by suicide. Moreover, suicide is a traumatic experience requiring specific help. In fact, being bereft by suicide is now understood to be a specific risk factor for a suicide attempt (Qin *et al.,* 2002; de Groot and Kollen 2013; Pitman *et al.,* 2016) making timely and specific intervention important.

None of the interviewed patients mentioned receiving support from their primary health care nurse. The primary health care team in Estonia comprises 1–2 nurses working with one GP; furthermore, the primary health care team is the first contact point for any health problem, serving as gatekeepers for the health and wellness needs of a unique patient panel (Põlluste and Lember, 2016). The restructuring of health care delivery relies on a team approach to serving the needs of patients, a shift that is described in a recent ‘Position Paper on Mental Health’ which mentions the importance of teamwork in primary care to meet the complex needs of patients struggling with suicide bereavement (Smit *et al.,* 2020).

Our participants valued help from the psychologist and the psychiatrist, especially in the early phase of grief. Some participants also reported that support groups were a valuable resource. This finding is confirmed by other studies which have found that suicide bereavement support groups are highly valued during this difficult time (Nic an Fhaili *et al.,* 2016). However, the research on this topic also cautions that evidence for the efficacy of suicide bereavement support groups is lacking (Cerel *et al.,* 2009). The systematic literature review by Linde, *et al.*, found bereavement groups to be effective in lowering the intensity of uncomplicated grief, but those with high levels of suicidal ideation should be engaged in cognitive-behavioral programs (Linde *et al.,* 2017). Based on the recent systematic review, interventions which show some effectiveness for people bereaved through suicide include supportive, therapeutic and educational approaches, and comprise a series of meetings led by trained facilitators; however, the methodological quality of available studies was poor (Andriessen *et al.,* 2019).

Our interviewees valued informal support systems, such as help from family members and friends. Also, previous studies have found that family and close friends are the most valued source of support (Pitman *et al.,* 2018). Interestingly, physicians who are bereaved by a patient who has completed suicide also seek informal support from their peers and colleagues rather than from formal services (Saini *et al.,* 2016). In addition to informal support, our respondents commented that general, practical information about grief and death is valuable. Unfortunately, it was not provided often or soon enough, leading to respondents having to search for answers alone while building up personal coping strategies. Self-care support should be offered more freely in primary care, but we recognize that this support must be preceded by sufficient training among clinicians and staff to recognize and refer patients with unmet bereavement needs. Through such outreach and support, we can help reduce inequities in recovery from trauma.

A focus group among individuals experiencing suicide bereavement found that people valued a proactive approach from a GP and that the physician should initiate the contact (Nic an Fhailí *et al.*, 2016; Pitman *et al.,* 2018). From the GP’s perspective, a qualitative study identified the pressure of time and low confidence in mental health care as the major barriers to offering help (Leavey *et al.*, 2017). Therefore, appropriate training should be provided to GPs and nurses regarding how to support those bereaved by suicide and how to include interdisciplinary (including mental health and social care professionals) teamwork possibilities more into primary care (Elzinga *et al.,* 2020). Although in-person visits are difficult during the present COVID-19 pandemic, telemedicine has been found to be quite able to meet the mental health need of patients (Torous *et al.,* 2020). Our findings suggest that GPs mostly offered a clinical diagnosis and antidepressants to patients bereaved by suicide. However, medication should not be the singular remedy offered (Nic an Fhailí *et al.,* 2016). Every GP should be provided with the knowledge and skills needed to proactively consult patients bereaved by suicide and offer a range of services and referrals.

### Strengths and limitations

We recruited people through media, the Internet and newspapers. Those who contacted us had a strong motivation to participate; thus, their experiences may not be readily generalizable to those who did not volunteer. Also, we were able to recruit only Estonian-speaking people therefore we excluded important demographics, such as the Russian-speaking population. One interviewer was also working as a GP (part-time), but there were no potential role conflict situations with respect to this researcher and her panel of patients.

The strength of our study is that we did not restrict our study population to parents or family members but included all those people who were interested in bereavement support and were motivated to share their experiences. The participants who took part were open to sharing their ideas and experiences, evidenced by the length of their interviews. Based on the feedback provided during a debriefing among the three interviewers, all subjects were thankful and seemed to be relieved after having the possibility to talk about their own experience.

This study contributes meaningful insights regarding the needs of those going through suicide bereavement, the resources which they find valuable, and their unmet needs. The central finding of this work is the potential for primary care to develop the capacity to meet the bereavement needs of patients in the practice who already have an established relationship with the practitioner. For example, the interviewees emphasized the convenience of regular contacts and the possibility to talk about their experience with someone who is known. For those who share the same practitioner as the victim, an added barrier which must be overcome is a potential sense of stigma and lack of trust in discussing the topic. Special training should be provided to accommodate this scenario and enable the practitioner to reach out proactively to the bereaved.

## Conclusion

Individuals bereaved by suicide need support, which was mostly found among friends and family members. Based on our results, also primary care providers have an opportunity to provide bereavement support among their patients. Better collaboration among the primary care team as well as continuing medical education regarding the needs of the bereaved could enable to proactively identifying and supporting those in need.
